# New Criteria of Indication and Selection of Patients to Cochlear Implant

**DOI:** 10.1155/2011/573968

**Published:** 2011-10-13

**Authors:** André L. L. Sampaio, Mercêdes F. S. Araújo, Carlos A. C. P. Oliveira

**Affiliations:** Cochlear Implant Center and Otolaryngology Head and Neck Surgery Clinic, University of Brasília Medical School, 70350766 Brasilia, DE, Brazil

## Abstract

Numerous changes continue to occur in cochlear implant candidacy. In general, these have been accompanied by concomitant and satisfactory changes in surgical techniques. Together, this has advanced the utility and safety of cochlear implantation. Most devices are now approved for use in patients with severe to profound unilateral hearing loss rather then the prior requirement of a bilateral profound loss. Furthermore, studies have begun utilizing short electrode arrays for shallow insertion in patients with considerable low-frequency residual hearing. This technique will allow the recipient to continue to use acoustically amplified hearing for the low frequencies simultaneously with a cochlear implant for the high frequencies. The advances in design of, and indications for, cochlear implants have been matched by improvements in surgical techniques and decrease in complications. The resulting improvements in safety and efficacy have further encouraged the use of these devices. This paper will review the new concepts in the candidacy of cochlear implant. Medline data base was used to search articles dealing with the following topics: cochlear implant in younger children, cochlear implant and hearing preservation, cochlear implant for unilateral deafness and tinnitus, genetic hearing loss and cochlear implant, bilateral cochlear implant, neuropathy and cochlear implant and neural plasticity, and the selection of patients for cochlear implant.

## 1. Introduction

The first pediatric cochlear implant program was established at the House Ear Institute in 1980. Incredible as it may seem from the current perspective, the primary issue in that era was whether to consider implanting children at all. In 1980, the first child (a 9-year-old boy) was implanted, and by 1982, 12 children with age ranges from 3.5 to 17 years had been implanted in their program [1, 2] The House/3M device obtained Food and Drug Administration (FDA) approval for implantation in adults in 1984 and in children in 1986. In June 1990, the Nucleus-22 channel implant received FDA approval for implantation in children aged 2 years and older. Shortly thereafter, momentum gained rapidly, and by the mid-1990s, more children were being implanted than adults [2]. In the USA, the Food and Drug Administration, a consumer protection and healthy agency, has historically had great influence on cochlear implant candidacy. 

Numerous clinical trials have been conducted by the FDA since cochlear implants were first introduced, and numerous supplements have been submitted to the FDA as these devices have undergone technological improvements. Thus, the determination of the implant candidacy is ultimately based on the best knowledge and judgment of the managing physician. Changes in candidacy have primarily included implanting persons with increasing amounts of residual hearing, implanting persons with increasing amounts of preoperative open-set speech skills, implanting children at younger ages, and implanting greater numbers of persons with abnormal cochleae. 

The primary reason that selection criteria have changed is that patients with implants are obtaining increasing amounts of open-set word recognition with the available devises. Although this increased performance is largely due to the technological advancements that have occurred in the field, it may also due to, at least in part, to the fact that patients with greater amounts of residual hearing and greater amounts of residual word recognition skills are receiving cochlear implants. 

The candidacy requirements for pediatric, and adult, cochlear implantation have gradually loosened. The obvious goal is to never have a single patient perform more poorly with their cochlear implant than they previously performed with hearing aids alone [2–4]. Thus, with each relaxation in requirements, a waiting period is required to accumulate the data to demonstrate the degree of benefit obtained by implanting these patients with higher degrees of residual hearing [2]. To date, the most consistent finding is that patients with greater degrees of residual hearing preoperatively perform at higher levels with cochlear implants. In children, this situation is even more complex because of greater difficulty and reduced reliability of audiologic testing in very young children. Slowly though, the data that have accumulated support the implantation of patients at increasingly younger ages and in those patients with higher degrees of residual hearing. The indications for cochlear implantation have expanded, as many unilaterally implanted individuals are able to achieve open-set word recognition. Despite the benefits seen in unilateral implantation, many individuals have difficulty perceiving speech in noisy environments. Bilateral cochlear implantation has made great strides in providing individuals access to sound information from both ears, allowing improved speech perception in quiet and in noise, as well as sound localization. The purpose of this paper is to review the literature searching for the new concepts in the indication and selection of patients to cochlear implant. The following sections will deal with these new concepts separately.

## 2. Cochlear Implant in Younger Children

Widespread universal newborn hearing screening has led to increased identification of infants with hearing loss worldwide [5–7]. This increase in early diagnosis has led to greater opportunities for early intervention [6]. There is now abundant evidence that early implantation in children is advantageous [2, 4–6]. Language development in children begins to occur from birth and is nearly complete by the age of 6 years. Language skills, speech quality, and expressive and receptive vocabulary are enhanced by exposure to aural language from as early an age as possible. Data from Kileny et al. implant program clearly demonstrated that children implanted between the ages of 12 and 36 months outperformed children implanted between the ages of 37 and 60 months [5]. This, together with earlier identification of childhood deafness, is pushing the age at implantation lower.

For many years, the lower limit for age at implantation was 2 years. The first clinical trial to evaluate a multichannel implant in children was the Nucleus 22 device. The candidacy guidelines for this trial indicated appropriate candidates could be as young as 2 years of age. The first clinical trial to include children less than 2 years of age was with the Clarion device. The Clarion device could be indicated for children of 18 months if the Rx demonstrated ossification. All cochlear implant devices can be safely indicated for children of 12 months and older. As of 2000, the FDA has approved a device for implantation for those patients aged 12 months and older [4]. Although prior and ongoing research has attempted to address the unique issues of safety, candidacy, programming, and efficacy in this very young age group, CI in children younger than 12 months of age remains controversial 

Further reductions in age at implantation are currently limited by the nature of audiologic testing in very young children. In general, using modern techniques, a confident assessment of severe to profound sensorineural hearing loss can be made in a child by the time they are 12 months old. In cases of hereditary hearing loss or meningitis, a confident assessment can sometimes be made at ages younger than this. Some authors [5, 6] prefer to have children with probable severe to profound hearing losses referred to their program by the age of 6 months. In most cases this gives the program time to complete a thorough assessment with confidence and enables implantation at the age of approximately 12 months.

Inherent in the discussion of CI in children younger than 12 months is the ability to reliably diagnose children of this age group with bilateral profound deafness. With respect to diagnosis of infant hearing loss, a paramount concern is the issue of specificity: the risk of implanting a child without profound deafness. CI evaluation of a child younger than 1 year of age should include the following: an attempt at behavioral audiometry (i.e., VRA), bilateral OAEs, ear-specific and frequency-specific ABR or ASSR, bilateral tympanometry, and acoustic reflexes. Present OAEs with abnormal ABR or ASSR and absent reflexes should generate suspicion for AN/AD, and ECoG can be used as an additional test in this setting [5–7].

Speech perception testing in this age group remains challenging as most tests are language based and not appropriate for children younger than 1 year. use of the infant-toddler meaningful auditory integration scale (IT-MAIS), a parental survey of early speech development, has been employed reliably in this age group as a proxy for speech perception and linguistic development [3, 8]. This 10-question structured interview assesses the three categoriesof auditory-specific behavior, including vocalization, alerting to sound, and deriving meaning from sound [6, 8]. Parents score the frequency of various behaviors on a 4-point scale from never (0) to always (4). Normative is available for normal hearing children throughout the first year of life and older, beginning at 1 month old. The IT-MAIS survey can be administered to parents of children with suspected hearing loss and compared with age-specific norms in normal hearing infants.

Although used in a variety of research and settings, babble assessment is not currently a standard component of preoperative CI evaluation in children younger than 1 [6]. On the other hand, anesthetic risk is an important consideration for children younger than 1 year of age. Epidemiological studies of anesthesia-related complications have found the incidence of morbidity, mortality, and life-threatening adverse events in children younger than 12 months to be significantly higher than children older than 1 year of age [6, 9, 10]. 

Although anesthetic concerns unique to very young children exist, data in the CI literature support perioperative safety in the under-1 population. Lesinski-Schiedat et al. [11] reported no higher incidence of surgical complications in 27 children implanted under 1 year of age compared with older toddlers. James and Papsin [12] in 2004 analyzed inpatient records of 25 infants implanted between 7 and 12 months of age and found no anesthetic or immediate postoperative complications. More recent studies by Dettman et al. [13], Valencia et al. [14], and Miyamoto et al. [15] report no anesthetic or immediate postoperative complications in children younger than 12 months. Finally, in the largest series to date, Roland et al. [16] reported no immediate perioperative adverse events in 50 children implanted under 1 year of age. 

Even though the anatomical aspects of the facial recess and the cochlea are similar to older children, several risks are reported in the literature for cochlear implant in the younger group. Intraoperative blood loss, facial nerve anatomy, receiver/stimulator fixation, and device migration with skull growth present unique risks to children younger than 1 year [6]. Two sources of blood loss are important in very young children: bone marrow and emissary veins. Pneumatization of the mastoid bone increases with age, approaching 60% at 2 years of age. Very young children, therefore, have a greater proportion of bone marrow in their mastoid and greater risk of blood loss during mastoidectomy [6]. In addition, blood loss from mastoid emissary veins can have a greater impact on overall circulating blood volume in children younger than 1 year of age. [6]

Long accepted as a standard in temporal bone surgery, the use of facial nerve monitoring in CI surgery is nearly universal. In children younger than 1 year, minimal inferior extension of the postauricular skin incision, careful identification of the facial nerve, and judicious use of intraoperative facial nerve monitoring can assist with these surgical challenges. 

In very young children, the posterior scalp flap is typically thinner and more delicate, necessitating constant care and atraumatic handling of the skin flap and soft tissues [6]. A variety of device fixation techniques have been advocated in children, including creation of a bony well and suture tie-down or use of a tight soft tissue pocket without well or additional fixation [6].

The circumferential dural exposure for seating of the receiver/stimulator is preferable for some authers so as to minimize device profile and damage from external trauma [17]. Others advocate that the device stability achieved with a tight soft tissue pocket is equivalent to that accomplished by creation of a bony well while eliminating the risks of intracranial complications [18].

Ongoing advances in receiver/stimulator design, such as the recently introduced Nucleus 5 (CI512) series by Cochlear, attempt to address these issues by creating a thinner device contoured to the infant skull. The effect of this change in device design is as yet unknown [6]. Skull growth and electrode migration are also a consideration in this very young population. Infant head circumference undergoes a dramatic change in the first year of life, especially as compared with the rate of growth after 12 months of age 6. As mentioned previously, the cochlea is adult sized at birth and does not enlarge with age; however, with skull growth, the distance between the cochlea and the skull increases and may affect device movement [6].

Programing the cochlear implant in young children is another challenger. Optimal use of CI technology requires an accurate assessment of threshold and comfort levels, obtained in older children and adults with behavioral testing. As with diagnostic testing, behavioral methods are frequently inappropriate for the very young child [6]. Neural response telemetry of Cochlear Corporation (Sydney, Australia), neural response imaging of Advanced Bionics (Valencia, Calif, USA), and auditory nerve response telemetry of Med-El (Innsbruk, Austria) use an electrode in each array to record a response from the auditory nerve. Responses, termed electrically evoked compound action potentials (ECAPs or EAPs), can be recorded intraoperatively as well as in postoperative programming sessions [6].

In very young children, ECAP measurements obtained through NRT/NRI/ART have been used successfully as a basis for threshold (T) and comfort. In very young children, ECAP measurements obtained through NRT/NRI/ART have been used successfully as a basis for threshold (T) and comfort (C) levels in creation of initial speech processor MAPs [6, 19].

Many authors have provided evidence for long-term safety in the under-1 population. Roland et al. [16], 2009 reported one of the biggest series of young children implanted. A total of 50 patients implanted under 12 months of age were followed for up to 6.8 years postoperatively and demonstrated complication rates comparable with those of older children and adults. The authors reported 16% of complication and the predominance of minor complications in very young children group is probably due to highlighted issues of surgical technique, such as the importance of delicate tissue handling, careful surgical planning, and meticulous treatment of squamous epithelium.

At this point it is crucial to bring for discussion that CI in very young children is a safe and effective procedure. There is growing body of literature supporting improved auditory and linguistic outcomes in children implanted before 12 months of age [12, 13, 15, 16, 20, 21]. Taken together, early evidence suggests a higher rate of receptive and language development in children implanted under the age of 1. At present, outcome data in auditory perception and linguistic development suggest that early implanted children may be more likely to achieve their full potential and may reduce or eliminate the need for them to “catch up” or learn at a faster-than-normal rate to achieve age-appropriate norms [6]. 

In conclusion, CI in the very young child provides unique challenges in diagnosis and certainty of testing, anesthetic risk, surgical technique, intraoperative testing and postoperative programming, long-term safety, development of receptive and expressive language, and speech perception outcomes. Overall, research to date support-minimal anesthetic, surgical, and long-term complications, suggesting that early implantation poses minimal risk to children younger than 1 year of age. Benefit in areas of receptive and expressive language development and speech perception has been suggested by multiple studies.

## 3. Cochlear Implant and Hearing Preservation

This section will concentrate on research done in attempt to summarize strategies and results around the world in hearing preservation during cochlear implantation. This attempt has significantly changed the candidacy criteria in the last years [22]. A number of studies have shown that preserving residual acoustic hearing in the implanted ear is a realistic goal for many patients with severe high-frequency hearing loss. The addition of the electric stimulation to their existing acoustic hearing can provide enhancement for these patients. In addition, the preserved acoustic hearing can offer considerable advantages, as compared to a traditional cochlear implant, for tasks such as word recognition in backgrounds or appreciation of music and other situations where the poor-frequency resolution of electric stimulation has been a disadvantage [22]. 

The approach involves preserving existing residual acoustic hearing (low-frequency) in an ear to be implanted with a cochlear implant and then adding electrical stimulation via this cochlear implant for the missing high frequencies to produce speech understanding (and other hearing sensations) via combined acoustic and electric hearing (A + E).

Word recognition research in patients with varying degrees of hearing loss by Turner et al. [22] among others supports the fact that the place of articulation cue is difficult for most listeners with severe to profound high-frequency hearing loss, since substantial inner hair cell loss prevents the cochlea from transmitting the frequency cues (presumably via place coding to the brain conclusions. These regions of inner hair cell loss referred to as “dead regions”, and, has also it shown that word recognition is affected. Thus a severe to profound high-frequency hearing loss often cannot be successfully remedied by simple amplification [22].

More recently, however, preservation of residual acoustic hearing in the implanted ear has received attention. Preservation of hearing within the implanted ear has been reported in several animal studies from the 1990s. Ni et al. [23]. in 1992 found that placement of a short electrode in animals did not induce additional tissue damage distal to the region of the electrode. Xu et al. [24] in 1997 reported similar results. Maintenance of near-normal click-evoked ABR thresholds in the majority of cochleae in these studies suggested that hair cells, at least apical to the implanted electrode array, not only survive, but also can function at a near normal sensitivity. 

Hodges et al. [25] in 1997 reported that one-half of patients examined following implantation with a cochlear electrode had at least some behavioral response to acoustically presented tones. Von Ilberg et al. [26] in 1999 reported preservation of hearing in a human patient following implantation of a standard long-electrode device, and that, although poor, the residual acoustic hearing was capable of understanding speech above chance levels. Also in the late 1990s, the group of the University of Iowa began implanting a newly designed cochlear implant with a modified intracochlear electrode that was much smaller in diameter and 6 mm in length, in subjects with usable residual low-frequency hearing. Based on these early reports of preservation of hearing following implantation, a number of centers have specifically attempted to preserve residual hearing in implanted ears of their patients.

Some groups have attempted to preserve residual acoustic hearing using a standard-length electrode that is partially inserted into the cochlea, combined with “soft surgery” techniques designed to minimize trauma. Gstoettner et al. in 2004 [27] described full or at least partial preservation of hearing in 18 of 21 patients using this technique. Kiefer et al. in 2005 [28] reported that at least partial preservation of hearing was accomplished in 11 out of 13 patients. The mean threshold change for those 11 patients was approximately 15 dB at the lower frequencies, while the remaining 2 patients suffered essentially total losses. James et al. in 2005 [29] reported approximately an average of 25 dB loss in the lower frequencies for 12 patients implanted with a long electrode, including the data for two patients who suffered total losses. These same authors [29] followed up with additional data, showing that 10 out of 37 patients initially had residual hearing preservation immediately following surgery; several months later, this number had decreased to 7.

Poor-frequency resolution and pitch perception of the traditional cochlear implant can lead to deficits in music perception. While implant listeners generally are quite good at perceiving the rhythmic cues in music [22, 27], their recognition of melodies is usually much poorer than normal, especially when the rhythmic or lyrical cues are not available. The residual low-frequency acoustic hearing of A + E patients can provide assistance in pitch perception in these patients [22]. 

Gstoettner et al. [27] tested normal hearing traditional long-electrode, and Hybrid (short-electrode) patients on melody and instrument recognition and found that Hybrid patients were nearly as accurate as normals for melody recognition, whereas the long-electrode patients performed very poorly. For instrument recognition, the Hybrid patients did show a deficit compared to normals, but this was primarily for instruments in the higher-frequency ranges, where signal was transmitted via the cochlear implant rather than the acoustic hearing.

The preservation of residual hearing has been shown to be practical and effective solution for severe high-frequency hearing loss [22]. It can overcome some of the inherent disadvantages of traditional, electric-only, long-electrode cochlear implantation. These advantages of the A + E approach are primarily a result of the better frequency resolution provided by the residual hearing as compared to electric stimulation. Thus the advantages of the A + E approach are most evident in situations where frequency resolution is important, such as recognizing speech in backgrounds and music perception [22].

## 4. Cochlear Implant in Unilateral Deafness

Up to now, treatment modalities of unilateral deafness consist of no treatment, conventional contralateral routing of signal (CROS), or bone-anchored hearing aid (BAHA) hearing aid. Cochlear implantation makes a new treatment modality available for patients with single-sided deafness. Arndt et al. [30] have recently performed a study in eleven adult subjects with unilateral deafness of various causes. The aim was to evaluate the use of unilateral electrical stimulation with normal hearing on the contralateral side and after a period of 6 months compared with the preoperative unaided situation, conventional CROS, or BAHA hearing aids. All subjects were fitted in random order with a BAHA Intenso mounted on the soft band/tension clamp or with a CROS hearing aid. After test periods with both devices, the subjects received a CI.

The authors found that cochlear implantation improved hearing abilities in people with single-sided deafness and is superior to the alternative treatment options. The use of the CI did not interfere with speech understanding in the normal-hearing ear. Their data suggested that the binaural integration of electric and acoustic stimulation is possible even with unilateral normal hearing.

## 5. Cochlear Implant in Unilateral Deafness and Tinnitus

Tinnitus is a frequent and often debilitating condition, which is difficult to treat. The most frequently used therapies consist of auditory stimulation and cognitive behavioural treatment aiming at improving habituation and coping strategies. However, more causally oriented therapeutic strategies are lacking and need to be developed to relieve auditory perception disturbances. Even though the pathophysiology of tinnitus remains incompletely understood there is increasing evidence that tinnitus is related to alterations of neuronal functioning in the central auditory system. Like phantom limb pain, tinnitus as an auditory phantom perception seems to be the correlate of maladaptive attempts of the brain at reorganization due to distorted sensory input. This notion is confirmed by the finding that hearing loss is the most important risk factor for developing tinnitus and that most people with sudden unilateral deafness experience tinnitus. Animal experiments have demonstrated that reduced auditory input causes a dysbalance between inhibitory and facilitatory mechanisms throughout the central auditory pathways, which then results in reorganization of the tonotopic maps in the auditory cortex. This might represent a neuronal correlate of tinnitus [31]. Accordingly therapeutic strategies that either specifically compensate for hearing loss or normalize auditory input (e.g., hearing aids) have been shown to consistently attenuate tinnitus complaints [32].

In subjects who are deaf and who also have tinnitus in the affected ear, tinnitus treatments based on acoustic input are impossible. On the other hand, tinnitus suppression using electric stimulation has been reported to be successful. Buechner et al. [33] have initiated a study in order to investigate the potential of cochlear implantation (CI) in unilateral deaf subjects regarding tinnitus suppression, device acceptance, and restoration of spatial hearing. They studied five subjects with severe to profound unilateral deafness having also ipsilateral tinnitus. In monthly visits, the speech processor program was optimized, and the hearing performance as well as tinnitus was monitored. In addition, it was investigated whether the CI improves hearing in adverse listening situations when combined with the normal hearing side.

In 3 participants, the tinnitus was significantly suppressed while wearing the device. In the other 2 participants, the tinnitus could be reduced in certain situations. Speech perception tests revealed a significant benefit with the CI in combination with the normal-hearing side for 3 participants. All participants accepted the device in a clinical setting; adaptation of the frequency allocation was not required. They concluded that improvements were found regarding the hearing and the tinnitus. Not all participants benefit from the CI to the same degree and in the same situations. The results indicate that cochlear implantation in subjects with unilateral severe to profound hearing loss and ipsilateral tinnitus may be beneficial on a case-to-case basis. However, further work needs to be performed to define the appropriate indication criteria for selection of patients with unilateral deafness.

Kleinjung and colleagues [34] reported a case of a 55-year-old man suffering from severe right-sided tinnitus in consequence of sudden right-sided deafness. Multiple therapeutic efforts including intravenous steroids and tympanoscopy with grafting of the round window remained unsuccessful. One year after onset of symptoms, right-sided cochlear implantation was performed, which resulted in a complete abolishment of tinnitus after activating the implant. The authors posted that severe unilateral tinnitus after sudden deafness might represent a new indication for cochlear implantation 

The application of cochlear implants for tinnitus relief in patients with unilateral deafness has so far been described in another study [35]. All 21 patients included in that study had unilateral sensorineural hearing loss accompanied by severe tinnitus for at least two years. In 95% of patients beneficial effects could be demonstrated. Three patients showed complete tinnitus relief, whereas the majority demonstrated statistically significant improvement on tinnitus loudness and impact.

According to different pathologic changes that generate neural activity interpreted as tinnitus, there are several possible mechanisms which may account for tinnitus suppression after cochlear implantation. Some reports further support the model of tinnitus pathophysiology, in which chronic tinnitus as an auditory phantom perception might be the correlate of maladaptive attempts at cortical reorganization due to peripheral deafferentation [36]. As a consequence of this theory, restoration of peripheral sensory input may have long-term beneficial effects on tinnitus by plastic reorganization of the central auditory nervous system. Such a mechanism might be reflected by the observed time course in some patients where tinnitus improved over a period of about three months after implantation. Another possible explanation for the positive effect might be the masking of tinnitus following increased auditory information due to the cochlear implant. 

Residual inhibition might explain tinnitus suppression effects which outlast the active stimulation period for a certain amount of time. But some observation that tinnitus was neither perceived in quiet environments nor during sleep might not entirely account for this theory. The effect of the insertion of the electrode into the cochlear should also be discussed [36]. Cochlear implantation causes immediate and subsequent trauma to remaining cochlear structures. This might be of benefit in patients, in which abnormal activity of hair cells turns out to be a constant trigger mechanism for tinnitus. However, in these patients immediate postoperative effects due to destruction should be expected, which might occur independent of activation of the implant system.

In summary, disabling tinnitus resulting from sudden unilateral deafness should be considered as a new indication for a cochlear implant procedure. As demonstrated in this report and supported by the literature data, cochlear implantation may represent a chance for complete suppression of tinnitus in selected patients.

## 6. Genetic Hearing Loss and Cochlear Implant

The hereditary causes are responsible by greater than 60% of all prelingually deafened with environmental or iatrogenic causes responsible by the remaining 40% [37]. Some of them will have good performance with amplification, but a substantial number of patients will need cochlear implantation. The literature has been not so clear about the success of cochlear implantation in genetic hearing loss, because some series are composed by small number of patients.

### 6.1. Connexin 26 and 30

Connexin 26 (Cx26) mutation has been reported to cause 50% of cases of nonsyndromic autosomal recessive hearing loss which makes this disorder the most common cause of nonsyndromic hereditary hearing loss. Dominant Cx26 mutations have been associated with syndromic disease with skin disorders such as keratitis ichthyosis deafness syndrome and palmoplantar keratoderma with deafness, and they are rare [38]. 

The connexins are proteins. Six containes assemble to form half a channel or connexon and two connexons together create an intracellular gap junction channel involved in electrolyte transportation keeping the potassium gradient within the spiral ligament and stria vascularis and communication between cells too. When these proteins present a defect may, occur potassium accumulation, and lack of recirculation is presumed to lead to hair cell dysfunction and degeneration [39]. Another role has been suggested for the Cx26 channels, so they may be responsible for calcium mobilization involved in cochlear physiology regulation. One of the most common mutation, for Cx26 is the 30delG, also known as 35delG because the deletion can occur anywhere within a stretch of six consecutive Gs [40], despite of the identification of more than 100 mutations for Cx26. A temporal bone analysis of a heterozygous Cx26 mutation demonstrated intact spiral ganglion cells, no neural degeneration, absence of hair cells in the organ of Corti, and agenesis of the stria vascularis [41], which may explain good performance after cochlear implants in these patients, since they have greater neural integrity of peripheral and central auditory systems [41, 42]. A study with the majority of patients being homozygous for 35delG showed better speech and language ability after cochlear implantation [43].

### 6.2. Usher Syndrome

Usher syndrome is one of the most common cause of deaf blindness in humans [44], and it is an autosomal recessive syndromic hearing loss characterized by sensory impairment of ears and eyes, which results in congenital sensorineural hearing loss with progressive retinitis pigmentosa, and posteriorly, retina degeneration, loss of night vision after 10 years of age, restriction of visual field and sometimes blindness in adolescence.

The literature describes three types of the Usher syndrome. The type I (USH1) is the most severe form of the Usher syndrome and well discussed, accounting for 30–40% and it is characterized by severe to profound congenital hearing loss, motor development delay in children, and progressive retinopathy with vision loss, decreased peripheral vision, and central acuity in the first decade of life leading to blindness by young adulthood. Molecular studies have shown that the defective proteins are located within the developing auditory hair bundle, either within the stereocilia or kinocilium. The defective proteins in USH1—myosin VIIa, PDZ-domaincontaining protein harmonin, cadherin 23, protocadherin 15, and the scaffolding protein Sans—are hypothesized to be associated with hair bundle-linked-mediated adhesion forces [45]. 

In temporal bone examination from USH1 patients, severe degeneration primarily of the basal turn of the organ of Corti, atrophy of the stria vascularis, and a decrease in spiral ganglion cells were found. The cochlear neurons were diminished with an average of 68% neuronal loss compared with age-matched controls [45]. Patients have low-frequency residual hearing and little benefit from amplification so cochlear implantation is done as soon as possible because vision impairment makes sign language a temporary solution. Liu et al. [46] and of Blanchet et al. [47, 48] concluded that early implantation is critical to developing effective oral-auditory skills prior to visual loss, so the best results are seen in children implanted before 3 years of age. The best prognostic factor after cochlear implants seems to be the age implantation and not genotypic mutations. Other benefits of cochlear implantation like better quality of life and independent living are also well described [49].

### 6.3. Mitochondrial Disorders

Mitochondrial DNA (MtDNA) mutations are maternally inherited predominantly, because mitochondria are located at the cytoplasm, and the fertilized embryo receives almost only the ovum cytoplasm. The mitochondria is involved in adenosine triphosphate (ATP) production through oxidative phosphorylation process that is critical especially for those organs with high metabolic needs. At the inner ear outer hair cells and the stria vascularis have high ATP demands. The hair cells rely on an appropriate endocochlear potential produced by the stria vascularis and its many Na^+^ K^+^ ATP pumps. It is hypothesized that mitochondrial dysfunction results in ionic imbalances, cell injury, and then death with concomitant hearing loss. The basal aspect of the cochlea, which is responsible for high-frequency hearing, requires even greater metabolic support. As a result, early injury to this area like in aminoglycoside ototoxicity or noise exposure results in the classical high-frequency hearing loss associated with mitochondrial dysfunction, which slowly progresses to affect other areas of the cochlea [50, 51], and at temporal bone studies it is possible to see decreased concentration of intact spiral ganglion cells, greater injury to outer hair cells versus inner hair cells, and progression of dysfunction from the basal aspect of the cochlea to the apex [52, 53]. 

Since sensorineural hearing loss (SNHL) is found in 42% to 70% of patients with mitochondrial disorders and cochlear implants are the treatment choice for a large number of them, it becomes relevant to look at this disease group. In these patients hearing loss is classified as syndromic: mitochondrial encephalopathy, lactic acidosis and stroke-like episodes (MELAS syndrome), maternally inherited diabetes and deafness (MIDD syndrome), the Kearns-Sayre syndrome and chronic progressive external ophthalmoplegia (CPEO) and nonsyndromic. The nonsyndromic SNHL is associated with ototoxic side effect of aminoglycoside antibiotics and the predominant mutation at maternally inherited MtDNA is A1555G in more than 50% of patients that present SNHL after aminoglycoside use. SNHL occurring in patients with the A1555G mutation without aminoglycosides exposure is reported, but it is milder. Residual hearing is found in patients with A1555G mutation associated with lower thresholds for electric promontory stimulation that suggests spiral ganglion cell preservation, making them good candidates for cochlear implants. 

Usami et al. [54] related 14 cochlear implantees with A1555G mutation of them 13 had previous exposition to aminoglycosides and from all the patients with ototoxicity history that received cochlear implants 59% had A1555G mutation. Considering syndromic group, SNHL is found in 50% of MELAS patients, and A3243G mutation is responsible for about 80% of MELAS cases. The epidemiology of the A3243G mutation reveals a frequency of 16.3/100,000 in the general adult population. Cochlear implants in MELAS patients have been successful in treating SNHL A3243G mutation patients [55]. It is interesting to notice that MIDD can arise from the same mutation, but the phenotype is narrower. The SNHL presented by MIDD patients has been overcome successfully by cochlear implantation too. The Kearns-Sayre syndrome is a rare multisystem disorder in which we can see a slowly progressive disorder involving paralysis of the levator palpebrae, orbicularis oculi, and extraocular muscles and SNHL in at least 60% of KSS cases.

The A3243G mtDNA mutation, as well as more common large-scale deletions, has been found responsible in skeletal muscle biopsy specimens, and these are usually of sporadic origin in both KSS and CPEO [56], and both of them have showed good results with cochlear implantation [57, 58]. Other mutations or syndromes related to mitochondrial syndromic SNHL have no cochlear implants reported.

### 6.4. The Waardedenburg Syndrome

Waardenburg syndrome (WS) is an autosomal syndrome characterized by dystopia canthorum, hyperplasia of the eyebrows, heterochromia iridis, a white forelock, and variable sensorineural hearing loss present in 1 in 40,000 live births and responsible by 2–5% of all congenitally deafened children [59]. Four fenotypes are described as follows: type 1 (dystopia canthorum, sensorineural hearing loss, heterochromia iridis, white forelock, hypopigmentation, synophrys), type 2 (type 1 features without dystopia canthorum), type 3 or the Klein-Waardenburg syndrome (type 1 features plus hypoplastic muscles and contracture of the upper limbs), and type 4 or the Shah-Waardenburg syndrome (type 2 features associated with Hirschsprung's disease) [59]. In WS1 melanogenesis is affected by the action of PAX3 gene, a transcription factor.

Absence of melanocytes affects pigmentation of the hair, skin, and eyes as well as the neural crest cells that migrate and form the basis of the stria vascularis [60, 61]. Hearing loss has been noted in 35–75% of patients with WS1 and 55–91% of patients with WS II and may be due to lack of melanocyte pigmentation in the stria vascularis of the cochlea [61]. Temporal bone studies of WS patients have shown atrophy of the organ of Corti and the stria vascularis [61]. Treatment may involve amplification and cochlear implantation for profound sensorineural hearing loss. Studies of WS cochlear implants have demonstrated well performance in both closed and open-set word standardized tests [62, 63]. It is important to remember the increased incidence of auditory neuropathy in this patient population, which may undermine implant efficacy [64].

### 6.5. The Jervell and Lange-Nielsen Syndrome

The Jervel Lange-Nielsen syndrome (JLNS) is characterized by a constellation of syncope, sudden death, congenital sensorineural deafness, and cardiac arrhythmias like significant bradycardia, ventricular tachycardia, ventricular fibrillation, and torsades des points, which leads to syncope and sudden death if not treated [65]. 95% of these attacks were triggered by emotional stress, exercise, or loud noise, with sympathetic activation as a unifying theme. Genetic defect is located in the *KCNQ1* and *KCNE1* (LQT1) genes that form the slow component of the delayed rectifier potassium channel complex (90 and 10% of cases, resp.). 

This mechanism is responsible by endolymph potassium maintenance at the stria vascularis and ventricular repolarization by moving potassium ions out of the cell [66, 67]. Scala media and endolymphatic compartments of the vestibular end organs are found obliterated by the collapse of Reissner's membrane and membranes surrounding the saccule, utricle, and ampullae [68]. There are small series describing good results in auditory performance and speech intelligibility rating after cochlear implantation [69, 70], and all agree that with appropriate precautions regarding cardiac disorders, cochlear implantation may be performed safely in patients with JLNS allowing for improved audition.

## 7. Bilateral Cochlear Implant

Our auditory environments are noisy and full of multiple sound sources that are a challenge for auditory system. The binaural system is responsible for providing cues that segregate target signals from competing sounds, and it is able to identify sound sources in normal hearing listeners. Binaural hearing is the result of integration between inputs from the two ears and auditory pathways. The consequence of binaural hearing is the speech understanding when competing sounds are present, and it is well known that when listening with only one ear, sound localization becomes very difficult to achieve. Three primary effects on perception have been identified in binaural hearing: the head shadow effect, the binaural summation effect, and the binaural squelch effect [71]. 

The head shadow effect occurs during everyday listening conditions when speech and noise are spatially separated. For example, background noise coming from the right side would interfere with the right ear but the head would block (create an acoustic shadow) some of the interfering noise from reaching the left ear. Thus, the head shadow effect would result in a better signal-to-noise ratio (SNR) in the protected left ear [72]. A listener is able to selectively attend to the ear with the better SNR to improve intelligibility. The head shadow attenuates high-frequency sounds by approximately 20 dB but low frequency by only 3–6 dB [73], and this effect does not require central auditory processing. 

The head shadow effect has the largest impact on hearing with binaural cochlear implantation. The binaural squelch effect also operates when competing noise is spatially separate from the signal so that the two ears receive different inputs. Unlike the purely physical head shadow, however, squelch requires central auditory processing that integrates the signals from each ear so that the auditory cortex receives a better signal than could be possible from either ear alone [74]. The brainstem auditory nuclei process differences in timing, amplitude and spectral signals coming from the two ears, resulting in improved separation of speech and noise. Evidence of benefit of the binaural squelch effect is somewhat limited, not seen in all users, and is not as large as seen with the head shadow effect [72]. 

Binaural summation also refers to a central processing effect, but it is thought to occur when both ears are presented with a similar signal. The combined signals from the two ears are perceived as louder by up to 3dB compared with monaural listening to the same signal [75]. This doubling of perceptual loudness is accompanied by increased sensitivity to differences in intensity and frequency and can lead to improvements in speech intelligibility under both quiet conditions and when exposed to noise. The literature in this area is limited, and the benefit is not as great as the head shadow effect [72]. 

Considering the advent of cochlear implantation and the great benefits experienced by users, it is important to stress its limitations, some of them from the device itself and other ones associated to monoaural hearing. The unilateral cochlear implant user shows poor ability in sound source identification, and it could be difficult hearing speech in noisy environment, so if the bilateral cochlear implant recipients are able to utilize the effects described above, this ultimately determines the degree of additional benefit a second implant will provide. 

The literature now containes huge series of bilateral cochlear implantation, and authors reported the ability of bilateral CI users to hear speech in the presence of competing stimuli and they have advantage of spatial separation between target and competing speech [75–77]. A common finding of these studies was that binaural advantage occurred to a greater extent under noisy conditions than in a quiet environment with a better speech intelligibility, most of it because of head shadow effect. In a study conducted with 17 native English-speaking adults presenting postlingual deafness that received the same implant model (Nucleus 24 Contour implant) in both ears, either during the same surgery or during two separate surgeries, the authors found speech intelligibility improvement with one versus two implants improve with time, in particular when spatial cues are used to segregate speech and competing noise. 

Localization and speech-in-noise abilities in this group of patients are somewhat correlated [78]. In bilaterally implanted children, there is significant increase in speech discrimination when the authors compared bilateral implants with the best-performing unilateral implant [79, 80]. The William House group in the annual meeting at the September 15, 2007 Cochlear Implant Study Group (CISG) discussed the issue of bilateral implantation with about 250 CI professionals. 

The specialists were concerned about unilateral cochlear implanted difficulties in everyday listening conditions. Functional localization of sounds is not possible with only 1 implant, often creating a safety issue, and hearing in noise is very difficult. They consider bilateral cochlear implantation advisable based on the results of improved speech intelligibility and sound localization and the expansion of the receptive sound field. Finally they agree that both children and adult may have better auditory performance with bilateral implants compared with unilateral ones [81]. 

Bilateral cochlear implants may not be an option or recommended for all adult recipients. This could be due to health issues that prevent a second surgery, lack of insurance coverage, or, in many cases, a notable amount of residual hearing in the nonimplanted ear. In these cases, the use of a hearing aid in the nonimplanted ear can represent a viable, affordable, and potentially beneficial option for bilateral stimulation despite the asymmetry in hearing between ears. 

This asymmetry occurs because the type of auditory input received by the two ears is quite different. The cochlear implant provides electric stimulation, while the hearing aid provides acoustic stimulation, which in combination across the ears is likely to provide atypical interaural difference cues in time and level [82]. Performance in the bimodal condition was significantly better for word recognition and localization compared to the cochlear-implant only and hearing-aid only conditions as the authors could observe studying 19 adult patients which were implanted with Cochlear Nucleus 24 and worn Widex Senso Vita 38 hearing aid at the other ear [82].

### 7.1. Sequential or Simultaneous Bilateral Cochlear Implant?

When considering the best moment to do the second implant, surgeons should inform patients about the same risks from the first operation and that the additional benefits with the second one may be not so great (it only increases around 20%) [72]. This way, it seems that simultaneous than sequential binaural implantation is advisable due to avoidance of a second hospitalization and general anesthetic, and it will be possible to use the same processor for both implants with cost reduction. [83]. In children using bilateral cochlear implants (CIs), development of normal patterns of cortical activity occurs when interimplant delays are minimized. 

Gordon et al. [84] have examined data suggesting that after 3 to 4 years of bilateral CI use, normal-like patterns of bilateral cortical activity are promoted in children receiving bilateral CI with minimal interimplant delays and young ages but are not present in older children who had longer interimplant delays. The optimal binaural hearing has not been achieved yet with bilateral implants, probably because the processing strategies in use. Then some studies have been suggesting that separated processors may produce interaural cues that are inappropriate for the actual sound source, and the probable solution is the use of only one processor or coordinate processors.

## 8. Neuropathy and Cochlear Implant

Auditory neuropathy/dyssynchrony (AN/AD) is a form of hearing impairment characterized by moderate-to-profound sensorineural hearing loss, progressive or transient, in which function outer cells is preserved, but afferent neural activity in the auditory nerve and central auditory pathways is disordered [85]. The incidence of AN has been estimated as 10% to 14% of children diagnosed with severe to profound SNHL [85]. In the audiological evaluation, otoacoustic emission responses can be showing normal preneural cochlear activity found but evoked response from the auditory pathway is commonly absent. Cochlear microphonic responses (produced by polarization and depolarization of cochlear outer hair cells) are also presented.

The auditory pathway disorder is suggested by the absence or severe distortion of electric potentials from the auditory nerve (compound action potential) and auditory brainstem response (ABR) [86]. There are some hypotheses to explain AN/AD that might be produced by lesion in the cochlear inner hair cells, at the synapse between these cells and the Type1 auditory nerve fibers, and in the auditory nerve itself [85, 87]. Clinically patients present speech discrimination scores worse than predicted by pure tone audiology with poor response to hearing aid amplification [88]. 

The incidence of AN has been estimated as 10% to 14% of children diagnosed with severe to profound SNHL [89]. The natural history may be progressive or transient. Even in cases of mild hearing loss, those with prelingual onset often do not develop speech [87]. Hearing aids in these cases are rarely beneficial. However, cochlear implants are debated because if the site of lesion is the cochlea, then bypassing the inner hair cells with direct stimulation of the VIIIth cranial nerve should produce good results, but if the pathologic condition lies in the nerve itself, such as demyelinization of the VIIIth cranial nerve, then electrical stimulation might be expected to be subject to the same limitations as acoustic stimulation. Considering this second possibility, many clinicians have been very conservative about cochlear implantation as an option for auditory neuropathy. Buss et al. [90] reported results for 4 children with AN who had postimplant speech data comparable with the general pediatric population receiving implants. 

The Sydney Cochlear Implant Centre (SCIC) has one of the largest cohorts of pediatric patients with AN undergoing cochlear implantation in the world (*n* = 80). Many of the children have proceeded to successful implantation, with a smaller number failing to gain significant benefit [91]. Gibson and Graham [92] published an editorial about this theme in 2008 where they recommend first of all a detailed investigation of auditory neuropathy patients including electrocochleography, auditory brainstem responses, and electrically evoked auditory brainstem responses, together with imaging, in order to identify the site of the underlying pathological conditions that may produce the combination of otoacoustic emissions in the absence of auditory brainstem responses in children with hearing loss. It is suggested that in 75% of cases auditory neuropathy can merely be a result of surviving outer hair cells when inner hair cell function is compromised. 

The remaining cases of auditory neuropathy may have dysfunction of the afferent neural synapse, cochlear nerve, cochlear nucleus, auditory brainstem tracts, and central auditory system. They concluded that each case must be seen individually rather than continuing to use a misleading term. Teagle et al. [93] related the results of cochlear implantation in 37% of 140 children with AN diagnosis. Although 50% of the implanted children with AN demonstrated open-set speech perception abilities after implantation, nearly 30% of them with >6 months of implant experience were unable to participate in this type of testing because of their young age or developmental delays. No child with cochlear nerve deficiency (CND) in their implanted ear achieved open-set speech perception abilities. In a subgroup of children, good open-set speech perception skills were associated with robust responses elicited on electrically evoked intracochlear compound action potential testing when this assessment was possible. 

The authors suggest that variable performance after cochlear implantation observed here may be explained by a wide variety of impairments. Some children will not achieve benefit from implantation, probably because of a lack of electrically induced neural synchronization, the detrimental effects of their other associated conditions, or a combination of factors. Teagle et al. [93] considered the finding of central nervous system pathology at preoperative magnetic resonance imaging a poor prognosis for the development of open-set speech perception, particularly when CND is evident. During patient selection electrically evoked intracochlear compound action potential testing may help identify those children who will develop good open-set speech perception. Instead of recommending CI for all children with electrophysiologic evidence of ANSD, the stepwise management procedure described herein allows for the identification of children who may benefit from amplification, those who are appropriate candidates for cochlear implantation, and those who, because of bilateral CND, may not be appropriate candidates for either intervention. Auditory neuropathy is a complex multifactorial condition encompassing a spectrum of clinical syndromes and outcomes with cochlear implantation. 

It is common to find varied and diverse conditions associated with AN like prematurity, hyperbilirubinemia, other metabolic and genetic disorders, and infections, which may result in a range of clinical presentation from mild to profound SNHL associated with normal or abnormal inner ear structures and vestibulocochlear nerves [94].

## 9. Neural Plasticity and the Selection of Patients for Cochlear Implant

A study using functional magnetic resonance imaging (fMRI) [95] was conducted to examine the effects of deafness, age of language acquisition, and bilingualism by comparing results from normally hearing, monolingual native speakers, congenitally deaf signers of American Sign Language (ASL), and normally hearing, bilingual speakers who were native signers of ASL and speakers of English. In this comprehensive examination, a strong and repeated activation of the classical language areas of the left hemisphere was observed in normal hearing and deaf subjects, while processing their native language, English, or ASL. However, deaf subjects reading English did not display activation in these regions. These results suggest that the early acquisition of a natural language is important in the expression of the strong bias for these areas to mediate language, independently of the form of language. In addition, native signers—hearing or deaf—displayed extensive activation of homologous areas within the right hemisphere, indicating that the specific processing requirements of the language may also, in part, determine the organization of the language systems of the brain and support the hypothesis that the delayed and/or imperfect acquisition of a language leads to an anomalous pattern of brain organization for that language. 

The removal or inactivation of cochlear hair cells leads to an immediate loss of activity in the auditory nerve [96]. The structural and functional consequences in the central auditory system are strongly dependent on the age at which inner hair cell (IHC) inactivation or other deafferentation occurred, the time since inactivation, and the level of the auditory system [97]. In mammals, when the inactivation occurs before the onset of functional hearing, 50–90% of cochlear nucleus neurons will die within days of the inactivation [98]. Further substantial neuron loss occurs upstream, in the nuclei of the superior olivary complex [99]. However, just before the onset of hearing, IHC-inactivation dependent brainstem neuron death abruptly declines, suggesting a mechanism that is linked to the initiation of auditory function [98] and for which cellular and molecular mechanisms continue to be sought [100]. 

Physiologically, neurons that normally only receive excitation from a deafened ear become inactive following IHC inactivation at any age, but, paradoxically, those in other parts of the auditory system become hyperexcitable [101], a response that has attracted interest as a possible mechanism of tinnitus [102]. Partial lesions of one cochlea in adults result in a spreading cortical representation of sound frequencies originating from the undamaged portion of the cochlea [103]. Electrical stimulation may protect the developing auditory system from degeneration [104] because degenerative effects that follow IHC inactivation after the onset of hearing have been linked to the loss of afferent activity in the auditory nerve [105]. 

It is well known that auditory perception of cochlear implant users gradually improves after post-surgical activation, and this improvement occurs with variable extent in different age groups, over different time courses, and for different outcome measures [106]. The literature has demonstrated that in terms of speech development and language acquisition the best results come from children implanted under the age of 2 years very similar to normal-hearing children. However, the capacity for plasticity in adult sensory systems, given appropriate patterns of behaviourally significant input, has more recently become generally accepted [107]. 

## 10. Conclusions

In recent years the indication criteria for cochlear implantation (CI) have changed. To gain optimal benefits, early implantation in prelingually deaf children is necessary. Even additional disabilities are no longer contraindications for CI. Nowadays the criteria for implantation not only include deafness but also residual hearing. Combined electric-acoustic stimulation has been established as a treatment option in patients with hearing still functioning in the low frequencies. Due to the benefits of binaural hearing, bilateral CI has become standard over the last decade. Recent experience has shown the benefits of CI in unilateral deafness and in cases of severe tinnitus. The actual benefit of CI shows great interindividual differences. We usually expect (re-)habilitation of language communication skills with implantation.
